# An analysis of the readability characteristics of oral health information literature available to the public in Tasmania, Australia

**DOI:** 10.1186/s12903-016-0196-x

**Published:** 2016-03-17

**Authors:** Tony Barnett, Ha Hoang, Ashlea Furlan

**Affiliations:** Centre for Rural Health, University of Tasmania, Locked Bag 1322, Launceston, TAS 7250 Australia; Northern Dental Centre, Oral Health Services Tasmania, 2 Kelham St, Launceston, TAS 7250 Australia

**Keywords:** Oral health, Health education and promotion materials, Dental, Health literacy, Readability

## Abstract

**Background:**

The effectiveness of print-based health promotion materials is dependent on their readability. This study aimed to assess the characteristics of print-based oral health information literature publically available in Tasmania, Australia.

**Methods:**

Oral health education brochures were collected from 11 dental clinics across Tasmania and assessed for structure and format, content and readability. Reading level was calculated using three widely-used measures: Flesch-Kincaid Grade Level (FKGL), Flesch Reading Ease, and Simple Measure of Gobbledygook (SMOG) reading grade level.

**Results:**

The FKGL of the 67 brochures sampled ranged from grade 3 to 13. The grade level for government health department brochures (*n =* 14) ranged from grade 4 to 11 (5.6 ± 1.8). Reading levels for materials produced by commercial sources (*n =* 22) ranged from 3 to 13 (8.3 ± 2.1), those from professional associations (*n =* 22) ranged from grade 7 to 11 (8.9 ± 0.9) and brochures produced by other sources (*n =* 9) ranged from 5 to 10 (7.6 ± 1.5). The SMOG test was positively correlated with the FKGL (r_s_ = 0.92, *p <* 0.001) though consistently rated materials 2-3 grades higher. The reading level required to comprehend brochures published by government sources were, on average, lower than those from commercial, professional and other sources. Government materials were also more likely to contain fewer words and professional jargon terms than brochures from the other sources.

**Conclusion:**

A range of oral health information brochures were publically available for patients in both public and private dental clinics. However, their readability characteristics differed. Many brochures required a reading skill level higher than that suited to a large proportion of the Tasmanian population. Readability and other characteristics of oral health education materials should be assessed to ensure their suitability for use with patients, especially those suspected of having low literacy skills.

## Background

Information about oral health is provided in a variety of forms and obtained from a range of sources including the media and more directly from oral health practitioners. Verbal messages from a dentist may be reinforced through a printed handout or brochure provided to a patient regarding ongoing care. Most public and private dental clinics make printed information freely available to patients to promote oral health. It is important that printed educational material should be pitched at an appropriate level for a particular target audience and be “fit for purpose”. Research suggests however, that health educational materials are often designed at a level beyond that which could be readily understood by the average adult or the majority of the population [1].

Wilson [2] assessed the readability of 35 patient education information brochures used in community healthcare centres serving low-income populations. The author found that these were written at a level higher than that for an average patient to understand [2]. Alexander [3] assessed the reading level of 24 general dental educational materials and reported that over 40 % of them were written at a grade level higher than recommended. Hendrickson and colleagues [4] assessed readability as well as thoroughness, textual framework, and the terminology used in 27 paediatric oral health materials and found both conflicting information and variation in readability across publishers [4]. Noticeably, there has been limited research published on the readability of print based oral health information made available in Australia. Our search recovered only three recent studies [5–7] which was limited to information about paediatric oral health.

Systematic reviews [8, 9] have demonstrated a significant association between socioeconomic status (determined by income, education and occupational background) and dental caries. There is a similar association between low oral health literacy and self-reported poor oral health [10–12]. Given that health literacy contributes to oral health literacy, it is of concern that the Australian adult literacy survey found that nearly 60 % of Australian and 63 % of Tasmanian 15-74 year olds were not able to demonstrate the minimum level of health literacy “to meet the complex demands of everyday life” [13]. Compared to other states, Tasmania’s population of around half a million persons is one of the least advantaged in term of socio-economic status. It is widely disbursed and ageing rapidly; factors that all contribute to poorer oral health outcomes [14]. Within this context, the aim of this study was to assess the readability characteristics of print-based oral health information literature available to the Tasmanian public. The results may assist the future development and use of these materials to better tailor them to the literacy level of a particular target audience.

## Methods

### Sampling

Oral health brochures readily available to patients were collected from a convenient sampling of public and private general dental clinics located in large and small towns in Tasmania. A member of the research team initially visited 7 clinics and collected all the materials that were made readily available to the public free of charge. A verbal request was made to another 4 clinics to mail all available oral health brochures to the research team. Collection ceased when no new brochure was identified in the yield from the last site sampled.

A total of 238 information brochures were collected and the following inclusion criteria were applied to each: written in the English language; aimed at patients and not health professionals/educators; oral health specific rather than general health or disease conditions that may impact oral health; and were in current use.

**Fig. 1:**
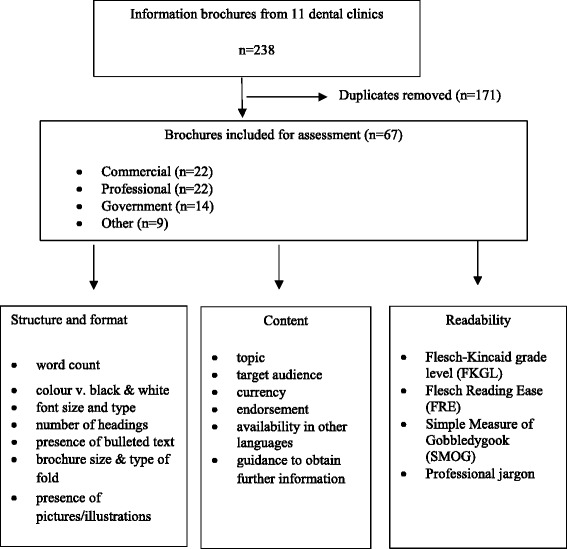
Design of the study

Figure 1 shows the design of the study. After removing duplicates, 67 (28 %) brochures remained and were grouped according to type of publisher: commercial (22), professional associations (22), government departments (14), and other (9) (eg. in-house) sources. The materials were assessed on 20 features across three major attributes: structure and format, content, and readability. Sampled brochures were scanned and converted to plain text Microsoft Word 2010 documents [15] for calculation of word counts and readability analyses.

## Structure and format

Presentation and layout of brochures can enhance readability and comprehension. Generally, materials that are complex, excessively wordy and or contain extraneous information are less likely to be read and can have less impact than those that are more focussed. Using everyday language, the logical sequencing of information, judicious use of white space, headings, bullets and using devices such as pictures or illustrations can help break up long passages of text, reduce text density and help reinforce key messages and assist understanding [16]. The structure and format of the brochures was evaluated by an assessment of: size of the brochure; type of fold (double fold, tri-fold or gate fold); the presence of bulleted text; number of pictures, use of colour, total word count; font size and type.

## Content

This attribute was evaluated using six features that included the broad purpose or content area of the brochure and target audience. Currency of information was assessed by locating the date on which the brochure was last published or reviewed. As a proxy measure for the accuracy and evidence-base of the information presented, we searched for any statement indicating endorsement by an appropriate (non-commercial) dental authority or professional association. Given that our sampling frame included clinics that serviced clients from different cultural backgrounds, we also looked for indicators that the brochure or the information it contained was available in languages other than English. Accepting that most brochures are designed to convey a particular message simply and succinctly rather than provide detailed or extensive coverage of the topic, we also looked for statements that directed the reader to additional sources of information on the topic.

## Readability

Readability refers to the degree to which written information can be understood by the reader. The reading level of each brochure was calculated using three widely-used measures. The Flesch-Kincaid Grade Level (FKGL) [17] and the Flesch Reading Ease (FRE) [18] score is calculated by formula based on average sentence length and average number of syllables per word. The Simple Measure of Gobbledygook (SMOG) is a count of polysyllabic words and typically used to analyse short documents [19]. The SMOG score may be calculated manually using a formula or via an on-line calculator [20]. Essentially, the more polysyllabic words, the higher the SMOG score.

Both the FKGL and the SMOG estimate the U.S. primary or secondary school grade level (from 1-12) that a reader would be expected to have completed in order to understand a particular text. These grade levels approximate those in the Australian school system. The FRE test produces a score from a given text that can range from 0 to 100. The higher the score, the easier a text is to read. In general, scores below 30 are “very difficult” and scores above 90 “very easy” to read [18]. The higher the FRE score, the lower the FKGL and SMOG grade level.

Professional jargon refers to words that would be uncommon to an average adult who had not received a health sciences education. Words that were explained in a brochure were not considered as jargon [4]. For this study, we drew upon lists of professional jargon identified by others [3-5] and extracted additional acronyms, dental or medical terms used in the sampled brochures that the research team thought could be difficult to understand. This list of 171 words was sorted alphabetically and five university qualified adults who were not health care professionals were asked to independently identify those words on the list that they found difficult to understand. Any word that more than one person rated as difficult was retained on the final list of 132 “professional jargon” words applied in our analysis.

## Statistical analysis

Data for each brochure were recorded in a spreadsheet then analysed using descriptive and inferential statistics available in SPSS 20. Descriptive statistics included, frequency tallies, range, mean, median and standard deviation were used to summarise characteristics related to the structure and format of each brochure as well as readability. A Spearman correlation coefficient was computed to determine the strength of the association between readability measures (Flesch-Kincaid, SMOG, and FRE).

Approval for the study from a research ethical review board was not required as our procedures did not involve the collection of data from humans or any experimental intervention. The study was restricted to a desk-top analysis of publicly available, print-based literature.

## Results

### Structure and format

Most brochures used standard paper sizes A3 (9), A4 (38) and A5 (2), though 18 were customised. A majority (60 %) of brochures were folded: 11 used a double fold, 21 tri-fold, 5 quad-fold and 3 utilised a gate-fold. The remainder (27) were single page hand-outs.

All except 4 brochures used colour and most (95 %) contained pictures or illustrations. The number of pictures used ranged from 0 to 17 (median: 5 pictures). A majority of brochures (79 %) distributed text under sub-headings (range: 13 (1-14), median: 4) and all but 1 made use of bulleted text. Brochures produced “in-house” were less likely to use pictures and more likely to be produced in black and white.

Word length ranged from 84 to 3856 (median 854 words). The smallest font size was 7 and largest 14. The most frequent font size used was 11 (used in 32 brochures). Ten different font types were used. The most frequently used was Calibri (22) followed by Gillsans MT (13) and DaxOT-light (10). Publishers tended to use one font type for their brochures. For example, Calibri was the preferred font type for all of the brochures sampled from the Australian Dental Association (Table 1).

**Table 1:** Structure, format and readability analyses of the brochures

Number	Publisher^a^	Type^b^	Title	Structure and format analyses						Readability analyses			
				Target audience	Word count	Font/size	Paper size	Fold	Number of pictures	Professional jargon (instances)	Flesh-Kincaid grade level	Flesh reading ease	SMOG reading level
1	Colgate	C	Oral health through everyday care	All patients	193	DaxOT-Light/10	A5	No fold	6	1	3.1	98.1	6
2	Colgate	C	Patient information: dental erosion	All patients	642	DaxOT-Light/10	A4	Trifold	5	1	9.1	54.3	11
3	Colgate	C	Oral health for teens and 20s	Teens and 20s	1759	DaxOT-Light/12	Customised	Closed gate	17	1	8.8	58.3	11
4	Colgate	C	Patient information: caries free teeth for a healthy smile	All patients	1032	DaxOT-Light/10	A4	Trifold	5	2	7.8	63.8	11
5	Colgate	C	Patient information: oral health for children 3-12	Parents	1471	DaxOT-Light/10	Customised	Closed gate	15	2	7.1	69.3	10
6	Colgate	C	Oral health and diabetes	Diabetic patients	934	DaxOT-Light/10	A4	Trifold	2	4	6.2	58.4	9
7	Colgate	C	Helpful tips to keep your gums healthy	All patients	787	DaxOT-Light/11	A4	Trifold	7	13	8.6	55.4	10
8	Colgate	C	12 h antibacterial protection against plaque	All patients	429	Calibri/7	Customised	Doublefold	7	6	8.8	53	11
9	Colgate	C	Sensitive teeth	Adult patients	728	DaxOT-Light/11	A4	Trifold	7	10	10.7	44.9	12
10	Colgate	C	Patient information: oral care during orthodontic treatment	Parents	1088	DaxOT-Light/10	Customised	Quadfold	15	5	6.7	70.4	9
11	Colgate	C	Patient information: oral health for infants and toddlers	Parents	1449	DaxOT-Light/10	Customised	Quadfold	10	0	7.8	67.1	10
12	3M ESPE	C	Winning formula helps prevent tooth decay: clinpro tooth crème	All patients	528	HelveticaNeue-Condensed + FZKMUV/11	A5	Doublefold	4	8	8.4	59.8	9
13	Polident	C	Denture care information booklet	Denture holders	1039	StoneSans/10	Customised	Quadfold	9	0	5.3	74.9	7
14	GSK Pronamel	C	Protect teeth against acid wear	Adults	359	StoneSans/8	Customised	Quadfold	6	3	7.6	58.9	10
15	GSK biotene	C	Do you suffer from dry mouth	Adults	1108	StoneSans/11	Customised	Closed Gate	2	2	7.3	65.4	9
16	GC	C	Tooth Mousse	All patients	734	Avenir 35 Light/9	A4	Trifold	5	1	8	63	11
17	GC	C	GC tooth mousse FAQS	All patients	744	Avenir 35 Light/8.5	A4	Trifold	4	3	7.7	64.5	10
18	GC	C	GC tooth mousse for seniors	Seniors	815	Avenir 35 Light/9	a4	Trifold	9	4	11	46.7	12
19	GC	C	GC tooth mousse for adults	Adults	731	Avenir 35 Light/9	A4	Trifold	9	2	10.9	45.8	13
20	GC	C	GC tooth mousse for children	Parents	612	Avenir 35 Light/9	A4	Trifold	9	13	12.6	43.5	13
21	GC	C	GC tooth mousse plus	All patients	826	Avenir 35 Light/9	A4	Trifold	4	5	10.2	51.9	12
22	Align Technology	C	Straight Talk about cooked teeth: how to reduce your risk of periodontal disease	Teens and Adults	739	Avenir-Book/10	Customised	Quadfold	6	10	10.7	49.2	12
23	ANZAOMS	P	TMJ disorders	Adults	2701	Times New Roman/12	A3	Doublefold	2	18	9.9	51.8	12
24	ANZAOMS	P	Wisdom teeth	Adults	3155	Calibri/11	A3	Doublefold	4	15	7.5	65.6	10
25	ANZAOMS	P	Orthognathic surgery	Adults	3169	Calibri/11	A3	Doublefold	2	15	9.5	55.2	11
26	ADA	P	Root canal treatment	Adults	1834	Calibri/11	A4	No fold	4	15	9.8	56.4	11
27	ADA	P	Fissure sealants	All patients	1367	Calibri/11	A4	No fold	2	9	9.4	54.8	11
28	ADA	P	Treatment of gum infections	Adults	1520	Calibri/11	A4	No fold	2	7	9.7	54.9	11
29	ADA	P	Crowns and bridges	Adults	1474	Calibri/11	A4	No fold	2	7	8.7	60.9	10
30	ADA	P	Dental care for babies and young children	Parents	3447	Calibri/11	A3	Doublefold	6	18	7.6	66.8	10
31	ADA	P	Bisphosphonate treatment and oral health	Adults	1412	Calibri/11	A4	No fold	2	5	9.8	52.2	11
32	ADA	P	Disorders of the jaw joint	Adults	1181	Calibri/11	A4	No fold	1	9	9.9	53.4	11
33	ADA	P	Bruxism	Adults	1315	Calibri/11	A4	No fold	5	13	9	56.2	11
34	ADA	P	7 tips for healthy baby teeth	Parents	280	Calibri/7	Customised	No fold	1	4	8.7	62.1	10
35	ADA	P	Cracked tooth syndrome	Adults	1445	Calibri/11	A4	No fold	1	8	7.7	67.1	10
36	ADA	P	Orthodontics	All patients	1762	Calibri/11	A3	Doublefold	2	12	9.1	54.3	11
37	ADA	P	Dental implants	Adults	3459	Calibri/11	A3	Doublefold	6	4	9.3	55.6	11
38	ADA	P	Wisdom teeth	Late Teens and Adults	2925	Calibri/11	A3	Doublefold	4	10	6.9	68.8	9
39	ADA	P	Your oral health and smoking	Late Teens and adults	1416	Calibri/11	A4	No fold	5	9	9.1	57.6	11
40	ADA	P	Home dental care	All patients	3856	Calibri/11	A3	Doublefold	7	7	8	66.4	9
41	ADA	P	Veneers, bonding, bleaching and composite fillings	Adults	2785	Calibri/11	A3	Doublefold	9	7	8.6	60.4	12
42	ADA	P	Dental extractions	Adults	1833	Calibri/11	A4	No fold	1	9	8.6	60.9	11
43	ADA	P	Orofacial pain	Adults	1714	Calibri/11	A4	No fold	2	10	10.9	50.5	11
44	ADA	P	Amalgam fillings for teeth	Adults	1326	Calibri/11	A4	No fold	1	5	9.9	52.9	10
45	OHST	G	Tips for the first dental visit	Parents	145	Gillsans MT/11	Customised	No fold	3	0	5.8	74.8	8
46	OHST	G	Give your child’s teeth a healthy start	Parents	162	Gillsans MT/11.5	A4	Trifold	12	0	4.4	82.2	7
47	OHST	G	Free dental care for children and teens	Parents	168	Gillsans MT/11	Customised	No fold	2	0	4.5	80.3	8
48	OHST	G	Dental services for adults	Adults	339	Gillsans MT/11	Customised	No fold	1	0	6.8	66.3	9
49	OHST	G	Psst give my teeth a healthy start magnetic	Parents	114	Gillsans MT/10.5	Customised	No fold	10	1	5.1	73.4	7
50	OHST	G	Smoking and your oral health	Adults and Late teens	383	Gillsans MT/12	A4	Trifold	9	2	4.3	74.5	7
51	OHST	G	Caring for and cleaning your denture	Denture holders	436	Gillsans MT/11.5	A4	Trifold	7	1	5.7	70.4	9
52	OHST	G	Smoking? What’s happening in your mouth?	Late teens and young adults	150	Gillsans MT/12	Customised	No fold	7	1	4.6	81.6	6
53	OHST	G	Give your child’s teeth a healthy start: a guide for children 2–5 years	Parents	168	Gillsans MT/11	Customised	No fold	10	1	4.9	85.7	7
54	OHST	G	Give your child’s teeth a healthy start: a guide for children 12–24 months	Parents	204	Gillsans MT/10	Customised	No fold	13	1	4.4	78.1	6
55	OHST	G	Your child had fluoride varnish painted on their teeth today	Parents	81	Gillsans MT/14	Customised	No fold	2	0	4.3	82.6	7
56	OHST	G	Post op instructions	All patients	188	Gillsans MT/12	A4	No fold	1	0	5.2	76.8	8
57	Medicare	G	Child dental benefits schedule	Parents	618	Veranda/11	A4	Trifold	4	3	11.1	46.3	13
58	South Australia Dental Service	G	Sugar	All patients	660	Gillsans MT/11	A4	Trifold	5	2	7.5	66.4	9
59	Oral Health Promotion Clearing House	O	11 things you must know about protecting your teeth, gums and mouth	All patients	327	FranklinGothic-Book/10	A4	Trifold	5	1	9.8	56.7	11
60	University of Adelaide	O	Patient information pamphlet no. 9: early childhood decay	Parents	1105	FranklinGothic-Book/9	A4	Trifold	4	2	7.8	65.3	9
61	University of Adelaide	O	Patient information pamphlet no. 6: beating rampant decay	Parents	854	FranklinGothic-Book/9	A4	Trifold	3	2	7.4	65.4	9
62	University of Adelaide	O	Patient information pamphlet no. 2: tooth erosion	All patients	718	FranklinGothic-Book/9	A4	Trifold	3	1	8.1	63.3	10
63	University of Adelaide	O	Patient information Pamphlet no 5.: dental sealants	All patients	666	Arial/9	A4	Trifold	3	4	6.6	68.7	9
64	Private family dental practice	O	Information for patients interested in orthodontic treatment at Riverside family dental	All patients	508	Times New Roman/12	A4	No fold	0	1	9.1	57	11
65	Private family dental practice	O	Information for parents of children having a dental treatment under general anaesthetic	Parents	989	Times New Roman/12	A4	No fold	3		8.7	63.1	11
66	Private dental surgery	O	Home treatment after surgical dental extractions	All patients	231	Veranda/11	A4	No fold	0	0	6	71.8	9
67	Private dental surgery	O	Home treatment after simple dental extractions	All patients	193	Veranda/11	A4	No fold	0	0	5.1	74.7	8

^a^ *ADA* Australian Dental Association, *ANZAOMS* Australian and New Zealand Association of Oral and Maxillofacial Surgeons, *OHST* Oral Health Services Tasmania

^b^ *C* Commercial, *G* Government, *O* Other, *P* Professional

## Content

Table 1 shows that out of the 67 oral health brochures reviewed, 20 targeted adult patients, 21 all patients and 16 contained information about child oral health though this latter group targeted parents rather than children as the reading audience. Materials were also written for late teens and adults (6), denture holders (2), diabetic patients (1), and seniors (1).

A range of topics were represented in the brochures samples and included: preventative oral health care (9), care in response to a specific oral health condition (15), age group (13) or oral health specific to a general health status/condition (4). Seven were about oral health products and the remaining 19 focussed information on a dental appliances or procedures.

Only thirty one brochures (46 %) listed the year of publication or revision and these ranged from the years 2002 to 2014. Twenty eight brochures reported endorsement by a professional association, 14 by a government department and 20 did not list any endorsement. Thirty four brochures (51 %) provided information on how the reader could access additional information though there were only 6 brochures (5 from industry sources and 1 from a Government source) that were also available in up to 6 other languages.

## Readability

The FKGL of the 67 brochures ranged from Grade 3 to 13 (post-secondary). The SMOG test was positively correlated with the FKGL (r_s_ = 0.92, *p <* 0.001) though consistently rated materials 2–3 grades higher. The FRE scores ranged from 43.5 to 98.1 (the higher the FRE score, the more readable the material) and scores demonstrated a high inverse correlation with grades computed for the FKGL (r_s_ = -0.96, *p <* 0.001) and the SMOG (r_s_ = -0.90, *p <* 0.001).

The brochure produced by Colgate entitled “Oral Health through everyday care” had the lowest reading-grade level with a FKGL level of 3.1 and contained only1 professional jargon term (Table 1, #1). The highest reading level (12.6) required was obtained for a well-illustrated brochure “GC Tooth mousse for children” which had 13 professional jargon terms recorded (Table 1, #20).

Brochures produced by government publishers required a lower level of reading ability than those from other sources with median and mean scores for the FKGL and the SMOG being lower than those from commercial, professional and other sources (Table 2). The FKGL for government brochures (*n =* 14) ranged from grade 4 to 11 (mean = 5.6 ± 1.8). Reading levels for materials produced by commercial sources (*n =* 22) ranged from 3 to 13 (mean = 8.3 ± 2.1), those from professional colleges (*n =* 22) ranged from grade 7 to 11 (mean = 8.9 ± 0.9) and brochures produced by “other” sources (*n =* 9) ranged from 5 to 10 (mean = 7.6 ± 1.5).

**Table 2:** Descriptive statistics: selected attributes (*N =* 67)

Source	Commercial (*n =* 22)	Government (*n =* 14)	Professional (*n =* 22)	Other (*n =* 9)
Feature				
Structure and format				
Word count				
• Min-Max	193–1759	81–660	280–3856	193–1105
• Mean (SD)	852 (371)	272.5 (186.4)	2061.4 (949.6)	621 (329)
• Median	765	178	1738	666
Font size				
• Min-Max	7–12	10–14	7–12	9–12
• Mean (SD)	9.7 (1.1)	11.4 (0.9)	10.8 (0.9)	10.2 (1.3)
• Median	10	11	11	10
Pictures				
• Min-Max	2–17	1–13	1–9	0–5
• Mean (SD)	7.4 (4.0)	6.1 (4.1)	3.2 (2.2)	2.3 (1.8)
• Median	6.5	6	2	3
Readability				
Flesch-Kincaid Grade Level				
• Min-Max	3.1–12.6	4.3–11.1	6.9–10.9	5.1–9.8
• Mean (SD)	8.3 (2.1)	5.6 (1.8)	8.9 (0.9)	7.6 (1.5)
• Median	8.2	5	9.1	7.8
SMOG				
• Min-Max	6–13	6–13	9–12	8–11
• Mean (SD)	10.3 (1.7)	7.9 (1.8)	10.6 (0.8)	9.6 (1.1)
• Median	10.5	7.5	11	9
Flesch Reading Ease				
• Min-Max	43.5–98.1	46.3–85.7	50.5–68.8	56.7–74.7
• Mean (SD)	59.8 (12.2)	74.2 (9.9)	58.4 (5.6)	65.1 (6.1)
• Median	58.65	75.8	56.3	65.3
Professional jargon words				
• Range	0–13	0–3	4–18	0–4
• Mean (SD)	4.3 (4.0)	0.9 (0.9)	9.8 (4.2)	1.6 (1.5)
• Median	3	1	9	1

There were 132 dental jargon terms identified in the 67 brochures sampled (Table 3). The number used in each ranged from 0 to 18. Ten brochures (6 from government sources) contained no professional jargon terms. Government brochures (with the exception of #57 “Child dental benefits schedule”) used fewer professional jargon terms (mean = 0.9 ± 0.9) than those produced by “other” (mean = 1.6 ± 1.5), commercial (mean = 4.3 ± 4.0) or professional associations (mean = 9.8 ± 4.2). Brochures from these latter three sources also used a greater average number of words (Table 2).

**Table 3:** Professional jargon words

Professional jargon words (*n =* 132)				
Abrasive	CPPACP	Haemophilia	Microscopic	Pulpotomies
Abscess	Craze	Halitosis	MRI	Radiograph
Abutment	Crossbite	Herpes simplex	Nerve canal	Recede
Amalgam	Cusp	Hyperplasia	Occlusal	Remineralise
Anticariogenic	Decalcification	Hypersensitivity	Occlusion	Resin
Apicoetomy	Deciduous teeth	Impacted	Onlays	Resorption
Arch wire	Demineralise	Incision	Open bite	Retrognathic
Arthrocentesis	Denting	Incisors	Ophthalmic	Rheumatoid arthritis
Arthroscopy	Dentition	Inferior alveolar nerve	Orofacial	Root planning
Arthrotomy	Desensitising	Inlays	Orthodontics	Septicaemia
Atherosclerotic cardiovascular disease	Disc	Interdental	Orthodontist	Splints
Bio-available	Disclosing tablets	Intravenous fluids	Orthognathic	Strontium chloride
Biofeedback	Dissipate	Keloid	Osteoarthritis	Temporalis muscle
Bonding	Dormant	Lateral pterygoid muscle	Osteonecrosis	Temporomandibular joint
Bone graft	Dry socket	Lavage	Osteoporosis	Tooth crown
Bridge	Endocarditis	Leukoplakia	Paget’s disease	Tooth mousse
Bruxism	Endodontic	Lingual nerve	Periodontal	Tricalcium
Buropion	Erosion	Lustre	Periodontal ligament	Triclosan
Calculus	Expectorate	Malocclusion	Periodontitis	Trigeminal nerve
Canines	Fissure sealants	Mandible	Pharynx	Tubules
Caries	Fissures	Mandibular	Phosphate	Velopharyngeal
Cementum	Fluoroapatite	Masseter muscle	Post operative	Velum
Composite	Fluorosis	Maxilla	Potassium	Veneers
Condyle	Foramen	Maxillofacial	Prognathic	Xerostomia
Conjunctional tissue	Fossa	Melanosis	Propagating	
Connective tissue	Gingivitis	Mental Foramen	Prosthodontist	
Copolymer	Glass ionomer cements	Mental nerve	Pulp	

## Discussion

A number of factors contribute to the readability of health education materials such as content of the message, complexity, the language used, text type and size, visual appearance, layout and understandability [21]. In this study, we focussed upon the structure and format of materials, features related to content as well as readability metrics. The format and presentation of the brochures such as font type and size, use of headings, bullets and pictures as devises to illustrate key concepts and to break up passages of text can influence how readers engage with and comprehend the information presented [4]. In the present study, consideration had clearly been given to these (and other) factors by publishers though brochures from professional and to a slightly lesser extent those produced by commercial publishers tended to use a greater number of words and demonstrated readability characteristics suited to a more literate audience.

The median and most frequent font size used in the brochures reviewed was 11 point. Out of the 67 brochures, only 10 made used of a recommended font size between 12 and 14 points [21]. Noticeably, one brochure written for older people (Table 1, #18) used a font size of 9 points and could present a challenge for those with some visual impairment even with the assistance of glasses.

The reading level of brochures sampled ranged from grade 3 to grade 13. Within the context of the U.S. assessment of grade level, it has been recommended that the reading level of patient education brochures should be no higher than sixth- to eighth-grade [22, 23]. However, about 50 % of the 67 brochures sampled in this study were written at a level above this upper limit. This suggests that the information contained in these materials may be difficult to fully comprehend by sections of the Tasmanian population where it has been estimated that less than 50 % of people are able to demonstrate the minimum level of health literacy required to function adequately in this domain (skill level 3 or above) [13].

Consistent with the previous studies [4, 5], our findings indicated that government brochures were the easiest to read though did contain some jargon words and, as reported in an Australian qualitative study [7], could therefore still be confusing to a reader. Brochures from professional associations and commercial publishers made more frequent use of professional jargon. Such differences may reflect assumptions about differences in the overall literacy level of the populations serviced by each sector with government materials (public dental services) directed to a larger proportion of clients with lower levels of literacy.

Although a wide range of assessment tools is available, no gold standard has been established to assess the readability of print-based patient education information across all settings [24]. Generally, using more than one readability assessment tool is preferred over using a single measure. This can provide some assurance of reliability and also highlight different aspects of the attribute under investigation. In this study, we chose four indictors of readability for reasons of simplicity (ease to use) and their widespread use across health discipline areas: the FKGL, FRE, SMOG and count of professional jargon words [4]. Consistent with the literature, the FKGL, FRE, SMOG were highly correlated though the reading grade results obtained from the SMOG were consistently from two to three grades higher than results obtained from the FKGL. This was because the SMOG formula is based on 100 % comprehension ability i.e. stricter criteria [24]. For example, if a brochure has SMOG readability grade of 5, it indicates that all people with grade 5 reading skills would normally be able to comprehend the brochure.

Whilst strong associations have been demonstrated between literacy level and the health status of a population [25], health education and promotion materials are only one component of a broader public health message. Dental clinics often make use of oral health information materials to inform patients and augment verbal advice provided by the dentist and members of the dental team [26]. To help maximise their effectiveness, at the readability of these materials should suit the skill level and other characteristics of the patient. Identifying attributes important to the readability of materials can help dental practitioners apply the more relevant of these to the selection or even design of materials best suited to their patient’s needs.

Whilst our selection of printed materials was limited to one state of Australia and clearly not exhaustive, we did sample a relatively large number of brochures and, as found elsewhere [4, 5] they did vary in their readability characteristics. The readability attributes we assessed were limited. For example, we did not assess the use of active and passive verbs or directly measure white space, density or “clutter” in each brochure (however, our word, picture and heading counts provide some estimate of this). As acknowledged elsewhere [5] the list of professional jargon terms identified were subjective and, given the procedure we used, is likely to be an underestimate of the number of words someone with a low reading skill level would find difficult to read and understand. A critical observation to be made of this and similar studies is to recognise that reading skill level and the readability of health information is only one component of oral health literacy. The judicious and appropriate use of health education materials can however, contribute to public health and education measures directed to improving oral health outcomes.

## Conclusions

Print-based oral health materials provide important information about the maintenance of good oral health and prevention of disease. Oral health brochures are publically available for patients in both public and private dental clinics in Tasmania. However their reading characteristics differed. Government (health department) brochures were easier to read than those produced by commercial, professional and other publishers. Some brochures required a high reading grade level and may not be suitable for a wide range of patients. Readability and other characteristics of oral health education materials should be assessed to ensure their suitability for use with patients, especially those suspected of having low literacy skills. The criteria applied in this study could be used as a checklist when reviewing, selecting or developing oral health brochures.

### Ethics statement

This study did not involve human subject or animals. HREC approval was therefore not required.
